# Are evaluations in simulated medical encounters reliable among rater types? A comparison between standardized patient and outside observer ratings of OSCEs

**DOI:** 10.1016/j.pecinn.2023.100125

**Published:** 2023-01-29

**Authors:** Easton N. Wollney, Taylor S. Vasquez, Carolyn Stalvey, Julia Close, Merry Jennifer Markham, Lynne E. Meyer, Lou Ann Cooper, Carma L. Bylund

**Affiliations:** a Dept. of Health Outcomes and Biomedical Informatics, College of Medicine, University of Florida, Gainesville, FL, USA; b College of Journalism and Communications, University of Florida, Gainesville, FL, USA; c Dept. of Internal Medicine, College of Medicine, University of Florida, Gainesville, FL USA; d Dept. of Medical Education, College of Medicine, University of Florida, Gainesville, FL, USA; e Dept. of Hematology and Oncology, College of Medicine, University of Florida, Gainesville, FL, USA; f Graduate Medical Education, College of Medicine, University of Florida, Gainesville, FL, USA

**Keywords:** Graduate medical education, OSCE, Interpreter devices, Patient-clinician communication, GME needs assessments

## Abstract

**Objective:**

By analyzing Objective Structured Clinical Examination (OSCE) evaluations of first-year interns’ communication with standardized patients (SP), our study aimed to examine the differences between ratings of SPs and a set of outside observers with training in healthcare communication.

**Methods:**

Immediately following completion of OSCEs, SPs evaluated interns’ communication skills using 30 items. Later, two observers independently coded video recordings using the same items. We conducted two-tailed t-tests to examine differences between SP and observers’ ratings.

**Results:**

Rater scores differed significantly on 21 items (p < .05), with 20 of the 21 differences due to higher SP in-person evaluation scores. Items most divergent between SPs and observers included items related to empathic communication and nonverbal communication.

**Conclusion:**

Differences between SP and observer ratings should be further investigated to determine if additional rater training is needed or if a revised evaluation measure is needed. Educators may benefit from adjusting evaluation criteria to decrease the number of items raters must complete and may do so by encompassing more global questions regarding various criteria. Furthermore, evaluation measures may be strengthened by undergoing reliability and validity testing.

**Innovation:**

This study highlights the strengths and limitations to rater types (observers or SPs), as well as evaluation methods (recorded or in-person).

## Highlights

• Standardized patients rated learners more leniently than observers who used recordings.
• Observer and standardized patient ratings differed on 21 of 30 evaluation items.
• Findings suggest that ratings may differ based on modality and/or rater types.
• Mixed rater types may produce results more accurately reflecting learner skills.

## 1 Introduction

Objective Structured Clinical Examinations (OSCEs) are an important part of graduate medical education and residency programs wherein learners can practice clinical skills and be evaluated in a controlled environment reflecting real-world scenarios [[1], [2], [3]]. Skills evaluated during OSCEs include medical knowledge and interpersonal skills needed to facilitate patient-centered care [[2], [3], [4], [5], [6]]. Better interpersonal communication is associated with higher patient satisfaction and better patient-reported health outcomes [5,[7], [8], [9]].

OSCEs are focused on a variety of patient care issues and may include challenging clinical situations such as triadic interactions (e.g., clinician-patient-companion), using certified medical interpreters to communicate with Limited English Proficiency (LEP) patients, or interprofessional and team communication [[10], [11], [12], [13]]. Exams typically contain multiple stations where learners must effectively complete an interaction with a standardized patient (SP) (trained medical actor) within a predetermined time limit [2,14]. Exams may be scored in several ways, including by faculty or other outside observers. SPs are frequently asked to complete evaluations following an interaction [2] to provide a patient perspective.

Yet, several issues may hinder OSCEs ability to accurately evaluate learners. First, while OSCEs allow educators freedom to assess various skills, comparisons of OSCE ratings across institutions may be challenging due to the lack of standardized stations or evaluation measures, and existing measures may not be validated [2,4,15]. One systematic review found that limited evaluation measures evaluating learners’ communication skills exist, and measures that do include communication skills generally have not undergone validity and reliability testing [4,16]. As such, evaluation scores may not accurately measure the skills being assessed [4]. Second, issues may exist regarding the training of evaluators, and lack of appropriate training for SPs can further complicate issues related to validity and reliability of measures (e.g., inter-rater reliability) [16]. Currently, conflicting evidence exists regarding the reliability of OSCE scoring among SP evaluators. This is especially highlighted in the limited research comparing scores between SPs and outside observers, as it is unclear whether SP ratings are congruent with observer ratings [3,10,17,18]. Therefore, the overall goal of this study was to compare OSCE ratings between SPs and observers. The research questions were:

RQ1: Do professional observers differ from standardized patients in their evaluation of an LEP-focused OSCE station?
RQ2: What were the most divergent OSCE evaluation items between SPs and observers?

## 2 Methods

### 2.1 Data collection

Interns (n = 93) across 11 residency programs at the University of Florida College of Medicine participated in OSCEs over a one-week period toward the end of their first year of residency (March 13 to 18, 2019). Over the course of that week, interns were assigned 10 stations to complete, each within an 8-to-13-minute range. Our study coded interactions from one of the 10 OSCE stations (detailed below). All exams were recorded for educational purposes. This study received ethical approval (IRB201900860).

#### 2.1.1 OSCE certified medical interpreter station

During one OSCE station, interns were asked to communicate medical discharge instructions to a patient previously hospitalized for an infection that had developed at an incision site following surgery. The Spanish-speaking patient had Limited English Proficiency (LEP). The patient also had a companion with them who was bilingual in English and Spanish. However, interns were expected to follow protocol to communicate with the patient by using a certified medical interpreter (CMI) from a medical interpreter service provided by the hospital via the telephone in the exam room. A total of four SPs participated in the CMI station (two patient-companion dyads). The SP dyads at this station participated in 8 to 10 exams per day over a one-week period (M = 9.3).

### 2.2 Coding procedures

SPs had previously received training on the specific scenario they were to portray, as well as instructions for completing the evaluation form. In the exam room immediately following each learner interaction, SPs had a few minutes to complete an evaluation form on the computer containing 30 items. Questions on the evaluation included three sections: patient education, use of translation services, and professionalism/communication skills. Some questions included notes for SPs on how to score learners.

Two authors (ENW & TSV), acting as observers, independently coded the 93 interactions using video recordings of the interactions. Observers used the same evaluation instructions and measure as the SPs. Both observers initially coded a subset of the same OSCE recordings to establish inter-rater reliability (n = 27; Cohen’s Kappa = .73). Following, the two observers each coded a portion of the remaining recorded OSCE encounters.

### 2.3 Data analysis

To compare SP and observer evaluation scores, we conducted two-tailed t-tests to compare observer and SP scores for each of the 30 items on the evaluation.

## 3 Results

SPs gave significantly higher scores to the interns than observers on 21 items, whereas observers only gave higher scores to the interns than SPs on 1 item (see Table 1). On 8 items, there were no statistically significant differences between observer and SP scores.

**Table 1:** Descriptive representation of who scored residents higher during OSCE.

Scoring Item	SP Giving Credit More Frequently	Observer Giving Credit More Frequently	No Statistically Significant Difference
Objective Items – Patient Education			
The resident told the patient…			
1. that she had an infection at the site of surgery	X		
2. to take your medications exactly as directed			X
3. about possible medicine side effect of diarrhea or loose stools	X		
4. she should continue to use nicotine patch	X		
5. she should not start smoking again	X		
6. increase dose of hydrochlorothiazide from 12.5 to 25mg			X
7. take pain medicine (Percocet) if needed for pain			X
8. not to drive while using pain medicine	X		
9. not to take acetaminophen with pain medicine (Percocet)			X
10. to seek medical attention for worsening redness on the skin	X		
11. to seek medical attention if they have chills or fever	X		
12. to seek medical attention if they have increased pain	X		
13. to seek medical attention if they develop diarrhea or loose stools	X		
14. to follow up with the surgeon, Dr. Lincoln, next Monday at 2 p.m.	X		
15. whom to contact if they get worse			X
The resident…			
16. asked the patient if it is OK to talk in front of "other person in the room"	X		
17. addressed patient by name directly (can be in English or Spanish)	X		
18. introduced him/herself by LAST name and "doctor"	X		
19. explained that he/she was an intern or resident	X		
20. summarized the patient's discussion	X		
21. asked the patient to repeat back info that was discussed during consultation			X
Interpreter Services			
The resident…			
22. used the interpreter phone to discuss all medical issues.	X		
23. addressed the patient, not the interpreter.	X		
Subjective Evaluation Items			
The resident…			
24. avoided medical jargon	X		
25. maintained eye contact with the patient while using the phone		X	
26. used effective body language			X
27. expressed understanding of the patient's emotions	X		
28. listened carefully when the patient spoke			X
29. explained things clearly to the patient	X		
30. Overall, the resident treated the patient with courtesy and respect	X		

The most divergent evaluation items between SPs and observers included items such as whether the intern directly addressed patient by name in English or Spanish (item 17), *t*(92)= −3.66, *p* < .001, explained to the patient that they were an intern, (item 19) *t*(92)= −6.60, *p* < .001, summarized the discussion with the patient (item 20) *t*(92)= −4.89, *p* < .001, addressed the patient and not the interpreter (e.g., using pronouns such as “you” versus “she/he/they/the patient”) (item 24) *t*(92)= −5.66, *p* < .001), expressed understanding of the patient's emotions (item 27) *t*(92)= −3.66, *p* < .001), and treated the patient with courtesy and respect (item 30) *t*(92)= −7.67, *p* < .001). Table 2 includes a comparison of how scores of all 30 items differed between observers and SPs, including means, standard deviations, and t-test scores.

**Table 2:** Table of mean difference between SP and observer.

Scoring Item	SP	Observer	*t*(*df*)
Patient Education (Objective Evaluation Items)			
The resident told the patient…			
1. **that she had an infection at the site of surgery**	**.94 (.25)**	**.83 (.38)**	−3.01 (92)**
2. to take your medications exactly as directed	.94 (.18)	.90 (.24)	−1.72 (92)
3. **about possible medicine side effect of diarrhea or loose stools**	**.33 (.47)**	**.19 (.40)**	−3.87 (92)***
4. **she should continue to use nicotine patch**	**.67 (.47)**	**.16 (.37)**	−8.96 (92)***
5. **she should not start smoking again**	**.72 (.45)**	**.24 (.43)**	−8.01 (92)***
6. increase dose of hydrochlorothiazide from 12.5 to 25mg	.91 (.23)	.88 (.25)	−1.47 (92)
7. take pain medicine (Percocet) if needed for pain	.80 (.41)	.76 (.43)	−1.35 (92)
8. **not to drive while using pain medicine**	**.16 (.37)**	**.10 (.30)**	−2.52 (92)**
9. not to take acetaminophen with pain medicine (Percocet)	.16 (.37)	.15 (.36)	−.24 (92)
10. **to seek medical attention for worsening redness on the skin**	**.55 (.50)**	**.34 (.48)**	−4.32 (92)***
11. **to seek medical attention if they have chills or fever**	**.48 (.50)**	**.35 (.48)**	−3.140 (92)***
12. **to seek medical attention if they have increased pain**	**.65 (.48)**	**.44 (.50)**	−3.93 (92)***
13. **to seek medical attention if they develop diarrhea or loose stools**	**.23 (.42)**	**.08 (.27)**	−4.04 (92)***
14. **to follow up with the surgeon, Dr. Lincoln, next Monday at 2 p.m.**	**.92 (.26)**	**.76 (.34)**	−5.61 (92)***
15. whom to contact if they get worse	.70 (.46)	.67 (.47)	−.77 (92)
The resident…			
16. **asked the patient if it is OK to talk in front of "other person in the room"**	**.25 (.43)**	**.18 (.39)**	−2.16 (92)*
17. **addressed patient by name directly (can be in English or Spanish)**	**.97 (.18)**	**.81 (.40)**	−3.66 (92)***
18. **introduced him/herself by LAST name and "doctor"**	**.99 (.05)**	**.95 (.19)**	−2.81 (92)**
19. **explained that he/she was an intern or resident**	**.76 (.43)**	**.39 (.49)**	−6.60 (92)***
20. **summarized the patient's discussion**	**.41 (.49)**	**.15 (.36)**	−4.89 (92)***
21. asked the patient to repeat back info that was discussed during consultation	.27 (.45)	.25 (.43)	−.50 (92)
Interpreter Services			
The resident...			
22. **used the interpreter phone to discuss all medical issues**	**.91 (.28)**	**.88 (.32)**	−2.15 (92)*
23. **addressed the patient, not the interpreter**	**.94 (.24)**	**.70 (.36)**	−5.66 (92)***
Professionalism & Communication (Subjective Evaluation Items)			
The resident…			
24. **avoided medical jargon**	**.96 (.20)**	**.86 (.35)**	−2.57 (92)*
25. **maintained eye contact with the patient while using the phone**	**.75 (.43)**	**.86 (.35)**	2.42 (92)*
26. used effective body language	.95 (.23)	.92 (.27)	−.63 (92)
27. **expressed understanding of the patient's emotions**	**.80 (.27)**	**.67 (.30)**	−3.66 (92)***
28. listened carefully when the patient spoke	.99 (.10)	.98 (.15)	−.58 (92)
29. **explained things clearly to the patient**	**.98 (.15)**	**.86 (.35)**	−2.96 (92)*
30. **Overall, the resident treated the patient with courtesy and respect**	**.95 (.17)**	**.72 (.26)**	−7.67 (92)***

Note. For the evaluators, cell entries are means with standard deviations in parentheses. Two-tailed significance at *p* < .05*, *p* < .01**, *p* < .001***. Items bolded indicates statistical significance

## 4 Discussion

### 4.1 Discussion

Overall, SPs and observers differed significantly on most evaluation items (22 of 30 items). SPs were more likely to give learners higher scores than observers (21 items). Although most studies have focused on only one differing variable (e.g., scorer type or method), our study examined both different evaluator types (observers and SPs) and different evaluator methods (immediate in-person evaluation and video-recorded evaluation). In other words, evaluator type was tied to evaluator method in this study. We have contextualized our findings based on the broader literature that has examined different evaluator types (i.e., observers and SPs), or different evaluator methods (i.e., immediate in-person evaluation and video-recorded evaluation).

Our results conflict with one study that found observers to be more lenient in their scoring than SPs [10]. Our results also differ from similar studies that found no significant difference between observer and SP scores [3,19]. However, a study comparing evaluator methods (immediate post-encounter evaluations and video-recorded evaluations) found similar results; raters watching video-recorded evaluations tended to give lower scores. Specifically, evaluators using video-recordings to complete their evaluations were significantly more likely to correctly identify verbal and nonverbal cues, and observers were less likely to rate false positives [20]. This is similar to our findings regarding scoring differences of nonverbal communication items between observers and SPs and highlights the differences among evaluation methods, as SPs in our study did not have access to video recordings and had to rely solely on recall. Based on these findings, differences among scores could be due to access to video recordings as opposed to evaluator type (i.e., SPs or observers). However, because we did not control for any factors that could impact variability in scoring, future research should compare observer and SP ratings in the same setting or condition (i.e., all in-person exams or all video-recorded exams).

SPs’ decreased likelihood of accurately scoring nonverbal communication may be due to working memory, particularly when demands may be overwhelming (e.g., numerous evaluation items to be completed) [[21], [22], [23], [24]]. For example, one study found that SPs miss a significant amount of nonverbal behavior during observations, particularly when interactions require improvisation, such as the learner asking an unanticipated question not covered in SP’s training [21]. Condensed evaluation criteria may be one solution to increase SPs’ working memory during in-person evaluation by allowing for greater focus on fewer phenomena [[22], [23], [24]].

Though our study offers insight into the reliability of evaluation methods, there are also several limitations. Technical difficulties that learners sometimes experienced may have impacted this cohort’s scores on criteria related to the use of the interpreter service, as the hospital where data was collected now uses a different CMI system. This may especially impact results when compared to other cohorts who have used the updated CMI system. Studies may compare multiple cohorts’ OSCE scores who have used the same and different CMI methods or services to see how these scores may differ to account for any service-related issues.

### 4.2 Innovation

Our study offers an innovative approach by examining real world practice; SPs evaluating in real time versus observers evaluating after the exam. Previous studies have typically examined these issues separately (i.e., evaluator type or evaluation setting). For instance, several studies have compared scores of different evaluator types (SPs and observers) who completed evaluations at the same point in time (i.e., in-person following learner’s completion of the station) [3,19], and other studies have compared scores between in-person and recorded observations [20], although findings have been mixed. Tying evaluator type and evaluation method in our study allowed us to examine multiple aspects of evaluation simultaneously and discern discrepancies. This can inform future research on medical education and evaluation, although future research is needed to better understand which specific factors have the greatest impact on differing OSCE scores in controlled settings. Future research may use experimental or other controlled designs to best distinguish discrepancies, which may incorporate a combination of different evaluator methods (SPs and outside observers or faculty), and/or different evaluator settings (in-person and recorded observations). Lastly, we focused on a unique OSCE settings where learners had to communicate using a CMI for LEP patients, which creates additional communication barriers.

### 4.3 Conclusion

Our study found significant discrepancies between SP and observer scores, where observers were less likely to give learners credit for completing evaluation criteria. Using a mixed approach to rate evaluation criteria may be a novel and innovative solution, as there may be different strengths and weaknesses associated with each evaluation method or rater type. However, more research should be done to assess this. Furthermore, evaluation measures may also benefit from condensed criteria for raters to assess, particularly for in-person evaluators [21,22].

## Declaration of Competing Interest

The authors declare that they have no known competing financial interests or personal relationships that could have appeared to influence the work reported in this paper.
